# Assessing Caudal Epidural Anatomy in Children: A Comparison of Palpation and Ultrasound for Sacral Cornua Identification

**DOI:** 10.4274/TJAR.2025.251980

**Published:** 2025-05-30

**Authors:** Celal Kaya, Pınar Kendigelen, Ayşe Çiğdem Tütüncü, Güner Kaya

**Affiliations:** 1 İstanbul University-Cerrahpaşa Cerrahpaşa Faculty of Medicine, Department of Anaesthesiology and Intensive Care, İstanbul, Türkiye

**Keywords:** Analgesia, anatomy, caudal anaesthesia, paediatrics, regional anaesthesia, sacrococcygeal region, ultrasound

## Abstract

**Objective:**

The aim of this study is to compare the identification of the sacral cornua using palpation and ultrasound, and to evaluate the sacrococcygeal area via ultrasound across different age groups of children.

**Methods:**

This study included 348 children aged 1 to 84 months, who were divided into three age groups: 1-24 months, 25-48 months, and 49-84 months. Sacral cornua were assessed using both palpation and ultrasound imaging. Palpation findings were categorized as “good”, “difficult”, or “non-palpable”. Ultrasound imaging of the sacral cornua was classified as “clear”, “unclear”, or “invisible”. Measurements taken included the inter-cornual distance, the anteroposterior diameter of the sacral canal, the distance from the skin to the sacral canal, and the distance from the dural sac to the cornua level.

**Results:**

Palpation of the sacral cornua was rated as “good” in 75.9% of patients, “difficult” in 22.4%, and “non-palpable” in 1.7%. All patients with “good” cornua palpation were also classified as “clear” on ultrasound imaging. Among the cases with “difficult” palpation, 76% showed a "clear" ultrasound image, while 24% were “unclear”. Only one patient had “invisible” cornua on ultrasound. The mean distance from the dural sac to the cornua level was 3.72±1.64 cm, and this distance increased significantly with age (*P* < 0.01).

**Conclusion:**

Ultrasound is a valuable tool for identifying the sacral cornua, especially when palpation is difficult, and offers reliable, detailed information on sacral anatomy.

**Main Points**

• The study highlights that ultrasound is significantly more reliable than palpation in identifying sacral cornua. It offers clearer visualization, with nearly all showing identifiable cornua, compared to variable success rates with palpation.

• Younger children (1-24 months) tend to have less developed sacral structures, making both palpation and ultrasound more challenging compared to older age groups (25-48 months and 49-84 month groups).

• These findings have practical implications for anaesthetists performing caudal blocks. Ultrasound enables clinicians to accurately assess critical anatomical landmarks-such as the sacral cornua, sacrococcygeal membrane thickness, and the position of the dural sac-which change with age.

## Introduction

Caudal block is a frequently preferred intervention, mostly for subumbilical surgery, because it is an easily applicable method, guided by distinct anatomical structures. Technically, this involves palpation of both sacral cornua to identify the sacral hiatus, with the patient in the prone or lateral decubitus position.^1^

However, palpation may be challenging due to high body weight, young age, or anatomical variations. A caudal block can fail if the local anaesthetic is administered to the wrong sites, specifically penetrating the superficial soft tissue or entering the intravascular, intraosseous, or intrathecal areas. Such incidences, although rare, can have serious complications such as systemic toxicity or total spinal anaesthesia.^2, 3, 4^

Ultrasonography has emerged as a non-invasive imaging modality that enables real-time visualization and assessment of anatomical structures. This advancement has led to a notable increase in the utilization of ultrasound-assisted caudal blocks, aiming to enhance the safety and success of intervention.^5^ However, previous research indicates significant anatomical distinctions between paediatric and adult patients.^6^ These variances arise from the ongoing growth and development period in children, resulting in variations among different age groups.

In this study, our primary aim was to compare the methods of palpation and ultrasound imaging in the detection of the cornua, used to find the needle insertion site during caudal block. Secondly, the sacrococcygeal area was examined using ultrasound in both transverse and longitudinal sections in children aged 1 to 84 months, covering the age range most commonly targeted for caudal block procedures. This examination aimed to investigate anatomical variations across different age groups.

## Methods

**Figure 1:**
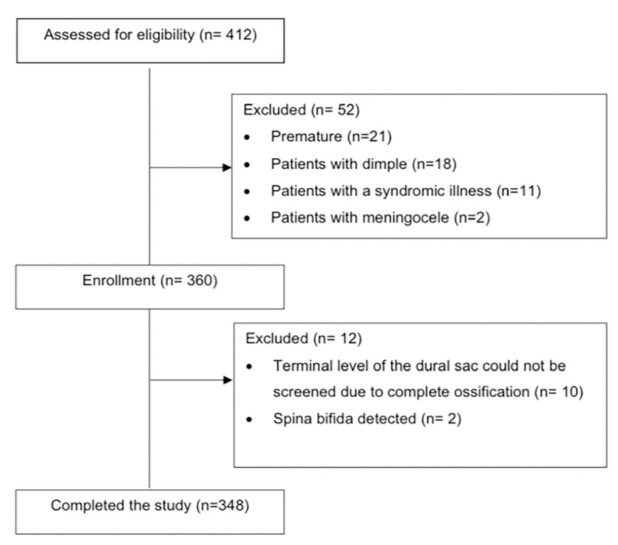
Flow diagram of the study.

Before patient enrolment, this prospective observational study was approved by the Clinical Research Ethics Committee of İstanbul University-Cerrahpaşa, Cerrahpaşa Faculty of Medicine (approval no.: 6470, date: 08.01.2019) and documented as no. 72109855-604.01.01- 103424, registered at clinicaltrials.gov (NCT03825172). Between January 2019 and January 2020, 348 children aged 1-84 months with American Society of Anesthesiologists I-II, who met the criteria for not having musculoskeletal, spinal anomalies, sacral dimples, history of prematurity, or a known syndromic illness were enrolled in the study after the written informed consent of the parents. Three age groups were formed as Groups 1, 2, and 3 to include, respectively, the 1-24 month, the 25-48 month, and the 49-84-month-old patients (Figure 1).

After induction of general anaesthesia, patients were placed in the lateral decubitus position with hips and knees in 90-degree flexion. The caudal area was then assessed through visual inspection and palpation. The cornua were classified as “good” when both cornua were easily palpable, as “difficult” if one or both were palpated with difficulty or one could not be palpated at all, and as “non-palpable” if both could not be palpated. Palpation was performed by two experienced anaesthetists, and classification was determined by joint consensus. Subsequently, the patients were examined using ultrasound to evaluate the sacral structures critical for the caudal block process.

**Figure 2:**
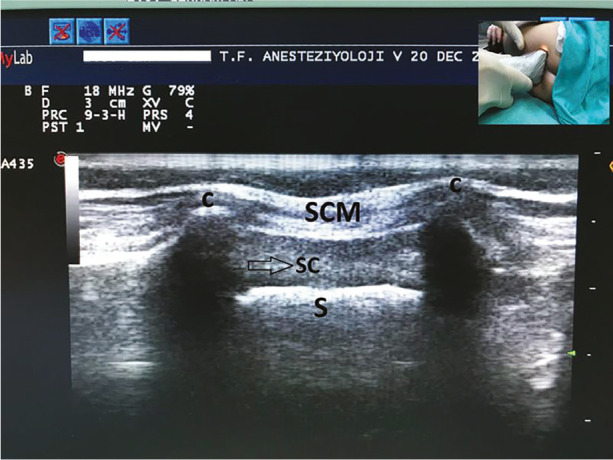
Transverse section of caudal ultrasonography at cornua level. C, cornua; SC, sacral canal; S, posterior wall of the sacrum; SCM, sacrococcygeal membrane.

All ultrasound investigations were carried out by the same anaesthetist in the presence of an observing anaesthetist with experience in the caudal applications of ultrasound. The ultrasound (Esaote Europe BV, Maastricht, The Netherlands) was used at a frequency of 12-18 MHz with the linear probe to visualize the caudal area. Firstly, a transverse section was obtained by positioning the probe on the spine over both cornua to capture the characteristic “toad face” image. The location was marked with a pen, and the following structures were identified (Figure 2).

Cornua: The transverse section was utilized to identify the highest points of both cornua, enabling the measurement of the distance between them.

If both shadows were clearly visible, the cornua were classified as “clear”. Weak unilateral or bilateral shadows, believed to correspond to the cornua, were classified as “unclear”. If no bone shadows were present, the image was categorized as “invisible”.

Sacrococcygeal membrane (SCM): SCM appears as a thin, shiny strip in the transverse section, covering the sacral hiatus of the posterior sacral canal. We opted to measure the distance from the skin to the epidural space instead of directly assessing the thickness of the SCM due to challenges in distinguishing it from the subcutaneous tissue in transverse sections.

Posterior sacral bone: In transverse imaging, it exhibits a distinct bright white appearance. It serves as the anterior wall of the sacral canal within this specific area.

The antero-posterior diameter of the sacral canal is the distance between the SCM and the posterior sacral bone measured on the transverse section.

**Figure 3:**
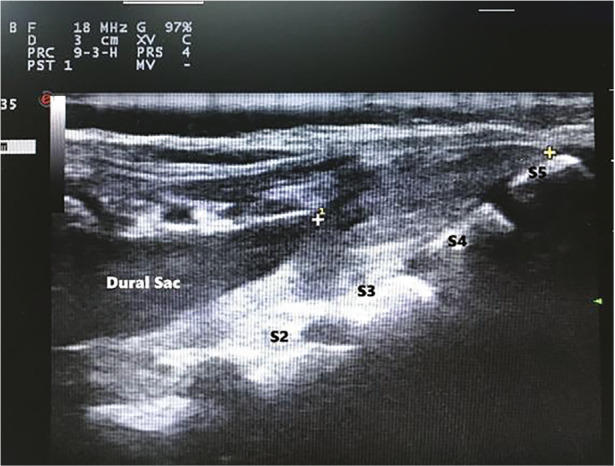
Longitudinal section of sacrococcygeal ultrasound. +…+ shows the distance between the terminal point of the dural sac and the cornua level.

Once the transverse section assessment was completed, the linear probe was rotated 90 degrees to obtain a longitudinal section. Subsequently, the dural sac, sacral epidural area, sacral vertebral bodies, intervertebral spaces and cornua level were clearly defined. The distance between the cornua level and the end point of the dural sac was measured (Figure 3).

### Statistical Analysis

The data were analyzed using the Statistical Package for the Social Sciences (SPSS) version 15.0 software (SPSS, Inc., Chicago, IL, USA). The descriptive statistics for the categorical variables were expressed in numbers and percentages, and the numerical variables were expressed in terms of the mean, standard deviation, the minimum and the maximum, the median, interquartile range, and the 95% confidence interval. As the numerical variables did not meet the assumptions for normal distribution, comparisons of more than two groups were carried out with the Kruskal-Wallis test. The subgroup analyses using a nonparametric test were made using the Mann-Whitney U test with the Bonferroni correction. The ratios in the groups were compared with the chi-square test. Since the relationships between the numerical variables did not meet the parametric test conditions, correlations were determined by calculating the Spearman correlation coefficients. A *P* value of <0.05 is accepted to indicate statistical significance. Based on Aggarwal et al.’s^7^ cadaveric study, which showed a bilateral cornua prevalence of 61.2%, the minimum sample size was determined to be 341 individuals, with 95% power and a 5% significance level.

## Results

This study was designed as a prospective observational with a planned duration of one year, based on our hospital’s patient records and estimated sample size. An extension was not necessary, as we were able to recruit a sufficient number of eligible patients within the one-year period. As seen in the flow chart, 412 children were assessed for eligibility. After applying the exclusion criteria, 360 children were enrolled for sacrococcygeal ultrasonography. In 10 children, the termination level of the dural sac could not be evaluated due to ossification, and in 2 children spina bifida was detected by ultrasonography. As a result, 348 children were included in this study (Figure 1).

The demographic and clinical data on the 348 children included in the study are shown in Table 1.

Table 2 summarizes changes in the palpability and ultrasound visibility of the sacral cornua across different age groups. Overall, palpation of the sacral cornua was rated as “good” in 75.9% of patients, “difficult” in 22.4%, and “non-palpable” in 1.7%.

The percentage of patients with “good” palpation was 65.4% in Group 1, 81.5% in Group 2, and 83.7% in Group 3. Group 1 had a significantly lower rate of “good” palpation compared to Groups 2 and 3 (*P* < 0.01), while there was no significant difference between Groups 2 and 3 (*P*=0.33). A statistically significant difference was observed overall across the three groups (*P* < 0.01). Only six patients had non-palpable cornua.

Ultrasound examination revealed that the cornua image was “clear” in 93.7% of all patients. Specifically, clear imaging was observed in 89% of Group 1, 96.3% ofGroup 2, and 97% of Group 3.

The rate of clear ultrasound imaging was significantly lower in Group 1 compared to Group 3 (*P*=0.04), with no significant differences between other subgroups. Only one patient had an invisible cornua image.

**Figure 4:**
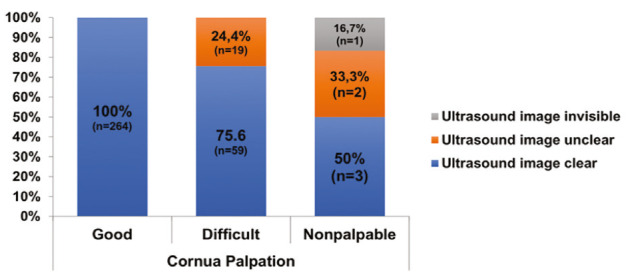
Ultrasound visibility of patients whose cornua palpation was classified as “Good”, “Difficult”, and “Non-palpable”.

All patients with “good” palpation (n=264) had “clear” cornua imaging on ultrasound. Among patients with “difficult” palpation (n=78), 75.6% showed “good” imaging, while 24.4% had “unclear” imaging. For those in whom the cornua were “non-palpable” (6 patients), 50% had "clear" ultrasound imaging (Figure 4).

Table 3 presents the ultrasonographic measurements of the caudal area in transverse and longitudinal sections, along with their variations across different age groups.

## Discussion

In the caudal block procedure, the epidural area is easily accessed through the sacral hiatus. This opening is formed by the non-fusion of the 5^th^ vertebral arches and in some cases, the 4^th^ sacral vertebral arches. Although caudal block is implemented quickly, and easily, the highly variable anatomical structure of the sacrum affects its safe application.^8^ In children, the distance between the dural sac termination point and the sacral hiatus is shorter than in adults, and the resultant proximity can cause accidental perforation of the dura.^9^ Understanding the anatomy of caudal block is critical for prevention of complications. Therefore, the anaesthetists have to determine the correct position of the patient and make the appropriate markings if necessary.

In an ultrasound-assisted study comparing straight position and flexion, it was shown that the dural sac progressed cranially with flexion. Positioning the patients with hip and knee flexion during caudal block was found to help the caudal needle reach the desired distance.^10^ Therefore, in this study, all children were placed in a standard position during the ultrasound imaging. The measurements were taken with hips and knees flexed at a 90-degree angle, a positioning commonly employed in caudal block procedures. Since urological surgery is more frequent with male children for reasons of circumcision or orchiopexy, the number of male patients exceeded the female patients in this study. However, Adewale et al.^9^ showed that magnetic resonance imaging of the sacral anatomy did not show significant differences between male and female children.

In children, ossification of the sacral bone is completed around 8 years old.^8^ Therefore, ultrasound can provide more detailed information in children compared to adults. After ossification is complete, it may not be possible to screen the lower level of the dural sac by ultrasound, hence, the upper age limit was set as 84 months. Ten patients near this upper age limit were excluded because the end of the dural sac was not visible on ultrasound. Additionally, palpation was done by 2 experienced anaesthetists to avoid any bias, and there was no conflict during the classification.

Although palpating the cornua to determine the sacral hiatus is a commonly employed method by clinicians, it is crucial to acknowledge that not all anatomical markers used to identify it are uniform across all patients. Sekiguchi et al.^11^ reported only being able to palpate 19 of the cornua in 92 adult cadavers, while Aggarwal et al.^7^ achieved bilateral palpation in only 30 out of 49 adult cadavers.In another cadaveric study by Aggarwal et al.,^12^ bilateral palpation was identified in 23 out of 39 fetuses (58.97%) that were at a gestational age of 7-9 months.

This underscores how ultrasound can be a viable alternative by considering the specific site of the sacral hiatus.

In our study, palpation of the sacral cornua was rated as “good” in 75.9% of patients, and when investigated by ultrasonography, it was found to be “clear” in 93.7% of these cases. Among patients whose cornua were rated as “difficult” to palpate, ultrasound revealed “clear” visualization in 75.6%, while 24.4% were “unclear”. Of the six patients with non-palpable cornua, three had “clear” visualization, two were “unclear”, and one was ‘invisible’ on ultrasonography (Figure 4). The effectiveness of ultrasound in imaging the caudal cornua was demonstrated by the fact that only one patient out of 348 had “invisible” cornua, leading to the assumption that this patient may not have developed cornua. Therefore, it can be concluded that in patients with developed cornua, ultrasound is unlikely to miss these structures. In summary, ultrasound proves to be a highly sensitive method for identifying the sacral cornua compared to palpation.

Additionally, there is greater difficulty in detecting the cornua by palpation and ultrasound in the 1-24-month age group (Table 2). Given that the bone structure of infants is not fully developed and anatomical markers may still be cartilaginous, some patients experience challenges due to the underdeveloped state of these structures and the presence of presacral fat tissue that may obstruct palpation. Analysis of ultrasound images by age group revealed that the percentage of “unclear” cornua was 10.3% in Group 1, decreasing to 3.7% in Group 2 and 2.9% in Group 3 (Table 2). This higher incidence of “unclear” images in infants likely reflects the incomplete development of the cornua in this age group. However, the percentage of “good” images remains high in Group 1, and given our experience with successful visualization in younger patients, ultrasound can be a valuable technique to guide anaesthesiologists during caudal block procedures.

In our study, the mean inter-cornual distance in the entire patient group was 1.19±0.11 cm, which was statistically less in Group 1, in comparison to the two older groups (Table 3). The shortness of this distance would make entry into the sacral canal difficult^13^, implying that manipulation of the needle is more difficult in younger age groups, including newborns.

The sacral canal should have the appropriate diameter for manipulating the needle with ease. Measurement of the antero-posterior diameter of the sacral canal during ultrasound imaging on the transverse section, gives important information to the physicians. Chen et al.^14^ measured the antero-posterior diameter of the sacral canal at the apex of the sacral hiatus and determined a mean value of 0.53±0.2 cm in 47 adult patients, who were injected caudally to treat sciatica pain during caudal ultrasonography on the longitudinal section. Despite previous findings indicating that the widest diameter of the sacral canal is at the upper section of SCM,^9, 12^ it is not always possible, as shown in this study, to obtain the image of the sacral hiatus apex on the longitudinal section in paediatric patients. Therefore, we have preferred to measure the antero-posterior diameter of the sacral canal from a specific image, obtained at the cornua level on the transverse section, simulates the “Toad” face (Figure 2). Therefore, our findings indicate a small sacral canal diameter. In our study, the mean value of antero-posterior diameter was 0.33±0.06 cm for all age groups. Interestingly, this distance was found to be wider in the second group compared to the other groups, which can be considered as an anatomical variability finding (Table 3). It is challenging to enter such a narrow canal with a needle. As a result, the frequent bone and subcutaneous tissue penetration may be a sign of narrower canals, which can be investigated with ultrasound.

The distance from the skin to the sacral canal is an important marker for directing the needle to the right point and ensuring a safe block. Very thin subcutaneous tissue can make it difficult to detect when the needle passes through the SCM, which would also complicate retracting the needle back to the subcutaneous tissue for adjustment to another angle. However, it is not always possible to differentiate the subcutaneous tissue from the SCM with ultrasound. Therefore, we opted to take these two structures together when making measurements (Figure 2). In our measurements, a notable and statistically significant increase in this distance was observed among older children (Table 3). This finding suggests a positive correlation between the child’s age and the combined subcutaneous and SCM thicknesses, indicating that as the child’s age advances, these thicknesses tend to increase.

One of the most feared complications of caudal block is penetration of the dural sac. Although rare, it can result in the life-threatening advent of total spinal anaesthesia.^2^ Such complications are avoidable since ultrasound enables detection of the position of the dural sac and the proximity to the injection site in children (Figure 3). In our study, the estimated mean distance between the termination points of the dural sac and the cornua level, which is considered acceptable for the site of injection, was 3.72±1.64 cm in children aged 1-84 months (Table 3). This measurement varied with age, such that the mean distance was 3.11 cm, 3.99 cm, and 4.30 cm, respectively, in Groups 1, 2, and 3. The differences were statistically significant (*P *< 0.01). These results indicate the necessity of care to prevent dural puncture in the infant group.

## Conclusion

This study demonstrates that ultrasound is a highly effective tool for identifying the sacral cornua in paediatric patients, especially when palpation is challenging. Compared to palpation, ultrasound provides superior clarity of the sacral anatomy, with nearly all patients showing clear visualization. Subsequently, inter-cornual distance, cornua level, combined subcutaneous tissue and SCM thickness, and distance between the dural sac level undergo modifications as individuals age.

## Ethics

**Ethics Committee Approval:** This prospective observational study was approved by the Clinical Research Ethics Committee of İstanbul University-Cerrahpaşa, Cerrahpaşa Faculty of Medicine (approval no.: 6470, date: 08.01.2019) and documented as no. 72109855-604.01.01-103424.

**Informed Consent:** Consent obtained directly from parents.

## Figures and Tables

**Table 1. Patient Characteristics:** 

-	**Total**	**1-24 m**	**25-48 m**	**49-84 m**
**Sample size, n, (%)**	348	136 (39.1)	108 (31.0)	104 (29.9)
**Age (months)**	30	11	34.5	64
**Median (IQR)**	(14-51)	(5-16)	(29-39)	(53.25-74.75)
**Sex, n (%)**				
Male	251 (72.1)	99 (72.8)	82 (75.9)	70 (67.3)
Female	97 (27.9)	37 (27.2)	26 (24.1)	34(32.7)
**Weight (kg)**				
Mean (SD)	13.8 (5.9)	8.9 (2.9)	13.9 (2.7)	20.3 (4.8)
(Min.-Max.)	(3-40)	(3-18)	(10-21)	(11-40)
**Height (cm)**				
Mean (SD)	92.5 (19.2)	75.2 (14.1)	95.6 (9.6)	112.1 (9.4)
(Min.-Max.)	(48-140)	(48-105)	(70-130)	(85-140)
**ASA classification, n, (%)**				
I	273 (78.4)	114 (83.8)	86 (79.6)	73 (70.2)
II	75 (21.6)	22 (16.2)	22 (20.4)	31 (29.8)

SD, standard deviation; IQR, interquartile range; ASA, American Society of Anesthesiologists; m, months; Min.-Max., minimum-maximum

**Table 2. Cornua Palpations, Ultrasonographic Evaluations of Cornua Positions and Changes with Respect to Age Groups:** 

-											**Subgroup analysis**		
**Group** **(age-month)**		**Total**		**1(1-24 m)**		**2(25-48 m)**		**3(49-84 m)**		-	**1-24 vs.25-48 m**	**1-24 vs. 49-84 m**	**25-48 vs. 49-84 m**
**Cornua **		**n**	**%**	**n**	**%**	**n**	**%**	**n**	**%**	***P* ^a^**	***P* ^b^**	***P* ^b^**	***P* ^b^**
**Palpation**	Good	264	75.9	89	65.4	88	81.5	87	83.7	<0.01^c^	0.01^c^	<0.01^c^	0.33
	Difficult	78	22.4	43	31.6	20	18.5	15	14.4	-	-	-	-
	Non-palpable	6	1.7	4	2.9	0	0.0	2	1.9	-	-	-	-
**Ultrasound image**	Clear	326	93.7	121	89.0	104	96.3	101	97.1	0.04^c^	0.07	0.04^c^	1.00
	Unclear	21	6.0	14	10.3	4	3.7	3	2.9	-	-	-	-
	Invisible	1	0.3	1	0.7	0	0.0	0	0.0	-	-	-	-

^a^Kruskal-Wallis test used to compare all of three groups; bSubgroup analysis with Mann-Whitney U test (Bonferroni correction P < 0.017); cChi-square test used to compare proportion.

m, month

**Table 3. Ultrasound Assisted Measurements on the Transverse and Longitudinal Sections:** 

- -		-	**Age**			-	**Subgroup Analysis**		
		**Total(n = 348)**	**1-24 m(n = 136)**	**25-48 m(n = 108)**	**49-84 m(n = 104)**	***P^a^***	**1-24 vs. 25-48 m *P^b^***	**1-24 vs.49-84 m *P^b^***	**25-48 vs. 49-84 m *P^b^***
**Inter-cornual distance** **(cm)**	Mean (SD)	1.19 (0.11)	1.13 (0.19)	1.23 (0.20)	1.23 (0.20)	<0.001	<0.001	<0.001	0.42
	Median	1.21	1.14	1.22	1.28	-	-	-	-
	IQR	1.06-1.33	0.98-1.26	1.08-1.36	1.12-1.37	-	-	-	-
	95% CI	1.17-1.22	1.10-1.17	1.19-1.26	1.20-1.27	-	-	-	-
**Antero-posterior diameter of sacral canal (cm)**	Mean (SD)	0.33 (0.06)	0.32 (0.05)	0.34 (0.06)	0.32 (0.05)	<0.01	<0.001	0.22	0.04
	Median	0.33	0.31	0.34	0.33	-	-	-	-
	IQR	0.30-0.36	0.28-0.34	0.31-0.36	0.29-0.36	-	-	-	-
	95% CI	0.32-0.33	0.31-0.33	0.33-0.35	0.31-0.33	-	-	-	-
**Distance from skin to sacral canal (cm)**	Mean (SD)	0.46 (0.14)	0.41 (0.11)	0.46 (0.13)	0.53 (0.16)	<0.001	0.03	<0.001	<0.001
	Median	0.44	0.395	0.425	0.505	-	-	-	-
	IQR	0.36-0.55	0.32-0.50	0.36-54	0.41-0.59	-	-	-	-
	95% CI	0.45-0.48	0.39-0.43	0.43-0.48	0.49-0.56	-	-	-	-
**The distance between the dural sac terminal point and the cornua level (cm)**	Mean (SD)	3.72 (1.64)	3.11 (1.53)	3.99 (1.97)	4.30 (0.95)	<0.001	<0.001	<0.001	<0.001
	Median	3.76	3.09	3.885	4.48	-	-	-	-
	IQR	2.90-4.25	2.36-3.76	3.28-4.21	3.81-4.88	-	-	-	-
	95% CI	3.54-3.90	2.85-3.37	3.61-4.37	4.10-4.49	-	-	-	-

^a^Kruskal-Wallis test used to compare all three groups

^b^Subgroup analysis with Mann-Whitney U test (Bonferroni correction) *P *< 0.017

SD, standard deviation; IQR, interquartile range; SCM, sacrococcygeal membrane
